# Artefacts and Family Resemblance

**DOI:** 10.1007/s13164-013-0145-4

**Published:** 2013-06-30

**Authors:** Pawel Garbacz

**Affiliations:** Department of Philosophy, John Paul II Catholic University of Lublin, Lublin, Poland

## Abstract

I develop in this paper a conception of artefacts based on L. Wittgenstein’s idea of family resemblance. My approach peruses the notion of frame, which was invented in cognitive psychology as an operationisable extension of this philosophical idea. Following the metaphor of life-cycle I show how this schematic notion of frame may be filled with the content relevant for artefacts if we consider them from the point of view of their histories. The resulting conception of artefacts provides a new insight into the current debate on artefact categorisation.

In the mid of the 1970s Nicholas Griffin made a sagacious observation on the fate of the idea of family resemblance. The gist of it has it that this idea had remained a philosophical programme without any realisation. No philosopher had bothered to employ it in order to analyse or describe any concept (Griffin 1974, p. 651). To my best knowledge, this finding has remained valid up to the present day provided that its scope is limited to philosophy. The conception of family resemblance has been quite popular, much debated, usually opposed to, but rarely, if ever, employed by philosophers. In my paper I intend to pick up this gauntlet for the (good) case of artefacts. Namely, I will build a concept of artefact in accordance with a particular interpretation of Wittgenstein’s idea and show how this concept may shed a new light on the problem of artefact categorisation.

Section 1 outlines the transition from the philosophical idea of family resemblance to its empirical validation in cognitive psychology. In particular, I sketch there the main features of L. Barsalou’s model of human concepts, which hinges upon the notion of frame. The next section employs this notion for the sake of the proper description of artefacts as objects-with-history. Section 3 shows the consequences of this description to the problem of artefact categorisation. The meta-theoretical status of my proposal is discussed in the last section.

## Family Resemblance: from a Philosophical Idea to a Psychological Experiment

Usually the idea of family resemblance is traced back to two quotes from Ludwig Wittgenstein’s *Philosophische Untersuchungen*:

Consider for example the proceedings we call games […] if you look at them you will not see something that is common to all, but similarities, relationships, and a whole series of them at that […] look for example at board games with their multifarious relationships. Now pass to card-games; here you find many correspondences to the first group, but many common features drop out, and others appear. When we pass next to ball games, much that is common is retained, but much is lost. Are they all amusing? Compare chess with noughts and crosses. Or is there always winning and losing, or competition between players? In ball games there is winning and losing, but when a child throws his ball at the wall and catches it again, this feature has disappeared […]. (Wittgenstein 1953)

(a) The tendency to look for something in common to all the entities which we commonly subsume under a general term. We are inclined to think that there must be something in common to all games, say, and that this common property is the justification for applying the general term “game” to the various games; whereas games form a family the members of which have family likenesses. […] The idea of a general concept being a common property of its particular instances connects up with other primitive, too simple, ideas of the structure of language. It is comparable to the idea that properties are ingredients of the things which have the properties; e.g., that beauty is an ingredient of all beautiful things as alcohol is of beer and wine, and that we therefore could have pure beauty, unadulterated by anything that is beautiful. (b) There is a tendency rooted in our usual forms of expression, to think that the man who has learnt to understand a general term, say, the term “leaf”, has thereby come to possess a kind of general picture of a leaf, as opposed to pictures of particular leaves. [.…] we are inclined to think that the general idea of a leaf is something like a visual image, but one which only contains what is common to all leaves. (Galtonian composite photograph.) This again is connected with the idea that the meaning of a word is an image, or a thing correlated to the word. (Wittgenstein 1958)

Both fragments, and also some others in which the idea of family resemblance is even less clearly alluded to, are not among the most perspicuous annunciations of Wittgenstein. Thus, it is little wonder that they gave rise to a number of interpretations. Most of them are composed of two parts. One is negative, but rather unambiguous: (at least) some concepts cannot be characterised by a set of conditions that are both sufficient and necessary. The other part is positive, but highly controversial: it states how it happens that a certain object falls under a family resemblance notion. In this paper I follow one of the first interpretations of the “positive part” by Ranford Bambrough from (Bambrough 1961).^1^ The idea of family resemblance is clarified here by means of the following example. Consider a family resemblance concept Φ. Suppose that Φ denotes a set of objects *a*, *b*, *c*, *d*. Bamborough distributes over these objects five properties: *A*, *B*, *C*, *D*, and *E*, which are supposed to somehow characterise Φ, as follows:

- object *a* has *B*, *C*, *D*, and *E*;
- object *b* has *A*, *C*, *D*, and *E*;
- object *c* has *A*, *B*, *D*, and *E*;
- object *d* has *A*, *B*, *C*, and *E*;
- object *e* has *A*, *B*, *C*, and *D*.

So although each object: *a*, *b*, *c*, *d*, and *e* is similar to any other by having at least one property in common, there is no property common to all of them. Bambrough concludes:

Here we can already see how natural and how proper it might be to apply the same word to a number of objects between which there is no common feature. And if we confine our attention to any arbitrarily selected four of these objects, say e d c a, then although they all happen to have B in common, it is clear that it is not in virtue of the presence of B that they are all rightly called by the same name. Even if the actual instances were indefinitely numerous, and they all happened to have one or more of the features in common, it would not be in virtue of the presence of the common feature or features that they would all be rightly called by the same name, since the name also applies to possible instances that lack the feature or features. (Bambrough 1961, p. 209–210)

Incidentally, Bambrough’s exposition allows also for an alternative interpretation of the negative part of Wittgensteinian conception: (at least) some concepts do not have the so-called characteristic intension – cf. (Ajdukiewicz 1974, p. 43–44) – i.e., if Φ is such a concept, then there exists no such set *X* of properties that (i) each object that falls under Φ has all properties from *X* and (ii) only objects that fall under Φ have all of them.

Even if Bambrough’s interpretation is not accurate to the initial idea of Wittgenstein, it is simple enough to be capable of invigorating research on concepts and of crossing over the border between philosophy and psychology. Although family resemblance still remains nothing but a programme *in philosophy*, it was successfully employed in psychology.^2^ Eleanore Rosch and Carolyn B. Mervis close their pioneering paper on the internal structure of human concepts with the following remark:

There is a tenacious tradition of thought in philosophy and psychology which assumes that items can bear a categorical relationship to each other only by means of the possession of common criteria attributes. The present study is an empirical confirmation of Wittgenstein’s (1953) argument that formal criteria are neither a logical nor psychological necessity; the categorical relationship in categories which do not appear to possess criterial attributes, such as those used in the present study, can be understood in terms of the principle of family resemblance. (Rosch and Mervis 1975, p. 603)

The hypothesis tested in (Rosch and Mervis 1975) can be summarised as follows. The subjects of their experiments were confronted with six classes of concepts with respect to which they were asked to make prototypicality judgments and/or describe these concepts by means of lists of attributes. The classes in question contain both “natural” categories, which correspond to the semantics of English words and phrases, and artificial categories, which were meaningless strings of letters and digits. Suppose that we define the similarity relation between such concepts in terms of overlap between those attributes: one concept is similar to another to the degree of the overlap between the attributes they have. The first part of the hypothesis in question posits that one concept is prototypical of another super-ordinate concept in proportion to the extent the former bears such similarity to other members of the latter. For instance, “chair” is a prototypical member of “furniture” to the extent the attributes of “chair” overlap with the attributes of other members of “furniture” such as “sofa” or “table”. The second part of the hypothesis has it that the most prototypical members have least similarity to concepts in other super-ordinate concept. Thus, the the attribute of “chair” should share less attributes with, say, “weapon” then, for example, with “sofa”. The six experiments described in (Rosch and Mervis 1975) proved these claims to be valid. As the reader may easily verify (Rosch and Mervis 1975) implicitly presuppose Wittgenstein’s notion of family resemblance along the lines established by Bambrough’s interpretation.

The results obtained by E. Rosch and her associates invigorated the research on the structure and dynamics of our conceptual life. Besides a number of specific experimental results three main types of theoretical models of concepts emerged: (i) prototype view, (ii) exemplar view, and (iii) theory view (Murphy 2002, ch. 3). These three models are theories with different predictive and explanatory capabilities, still they share some salient characteristics. Barsalou (1991) argues that there exists a distinct conceptual framework that unifies the main theories of concepts and whose capabilities are commensurable to the complexity of human knowledge. The main component of his proposal is the notion of frame, which is claimed to constitute a reasonable generalisation of the notion of attribute list.

A *frame* is an entity – (Barsalou et al. 1993) define it as a “data structure” – that is composed out of four types of components:

1. attributes
2. attributes’ values
3. structural invariants
4. value constraints.

**Fig. 1:**
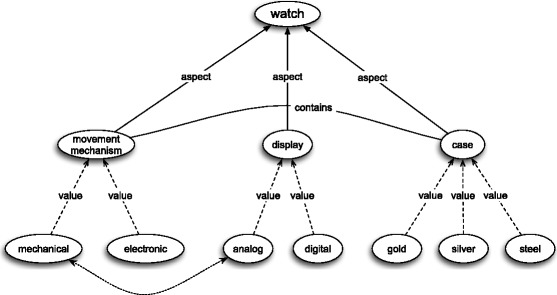
A frame for watch – example

The main role of frames is to represent concepts. Consider, for example, the concept of watch as represented by the frame depicted in Fig. 1. The frame describes this concept by means of three attributes: movement mechanism, display, and case. The movement mechanism attribute is to represent the physical principle of watch operation and our frame assigns it two values: mechanical and electronic. The display attribute characterises the ways in which standard watches provide time: analog or digital. The third attribute represents the kind of substance the watch’s case is made of. The frame in question also contain one structural invariant, which is represented by the solid line with the “contains” label on it. The role of this invariant is to integrate the case attribute with the movement mechanism: watch cases usually contain movement mechanisms. Finally, the frame contains also one value constraint – shown in Fig. 1 as an unlabelled dotted line – to the effect that mechanical movement co-occur with analog display. Generally speaking, each attribute of a concept represents a certain aspect or characteristic of the objects that fall under this concept.^3^ Each value of an attribute is a concept subordinate to this attribute. The structural invariants relate the attributes of the concept at stake and the value constraints relate the attributes’ values.^4^

Another specific feature of frames is their extendibility. Since (i) frames represent concepts and (ii) both attributes and their values are concepts, therefore there may exist frames that represent other frames’ attributes and values. Furthermore, there are frames for structural invariants and value constraints.^5^ Metaphorically speaking, there might be frames within frames.

One of the most both robust and intriguing finding in research on concepts and categorisation concerns the flexibility of concepts.

Consider the word “newspaper” and note which of its features come to mind. Now consider the word “newspaper” in the context of building a fire. Whereas feature *flammable* probably didn’t come to mind when you consider “newspaper” in isolation, it probably did when you considered it in the context of building a fire. Many researchers have implemented such manipulations in experiments and observed large effects on verification time. (Barsalou 1993, p. 31)

**Fig. 2:**
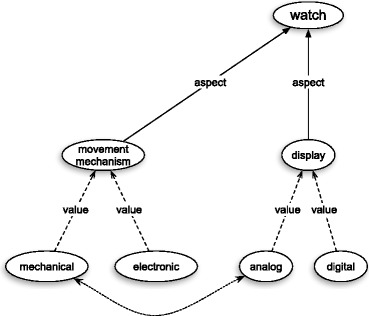
A variant for the main frame for the concept of watch

Generally speaking the flexibility of a concept consists in the variability of its content and structure with respect to different individual subjects who entertain this concept and with respect to different occasions (contexts) in which it is employed. The frame theory in question captures this phenomenon by means of what I call the main frame and its variants. According to the explanations given in (Barsalou 1992, p. 33–34) and in (Barsalou 1993, p. 33–34) people store in long-term memory a sort of maximal frame for each concept – the *main frame*. This frame is constituted by the most comprehensive set of attributes for this concept. Interestingly enough, quoting (Goodman 1955), Barsalou maintains that the latter set can be infinite. Barsalou (1993, p. 33–34) reports the results of an experiment that supports the claim that the content of the main frame is highly stable for individuals over extended periods of time. The main frame is accessed on different occasions by means of subsets of this set and each such subset may be represented by a frame – let me call it a *variant of * the main frame. For instance, if we make a (grossly oversimplified) assumption to the effect that the frame in Fig. 1 is the main frame for the concept of watch, then frame in Fig. 2 may be one of its variants. This means that if an object in front of me has, say, the mechanical mechanism and the analog display, but lacks a case, then it still falls under the concept of watch, even though it might not be a prototypical watch. On the other hand, if we made a (probably false) assumption to the effect that the frame in Fig. 2 is the only variant of the main frame for “watch”, then anything with an analog display and a golden case, but without any movement mechanism, would not be a watch.

Formally, the main frame for a concept can be represented as a directed connected labelled graph, with labels both on vertices and edges, and its variants as its connected subgraphs – see (Petersen 2007).

Barsalou (1991) shows how this conception of frames may be employed as a common conceptual framework for the classical view of concepts and its main non-classical competitors. Obviously, each of these views will use frames in a different way to express the internal structure of concepts, their role in categorisation, etc. In particular, the prototypicality predictions will differ among those views even if they are expressed by means of frames.

It is the idea of Barsalou frames that has shaped my proposal of a family resemblance notion of artefact.

## A Family Resemblance Notion of Artefact

The notion of artefact developed in this paper is based on the common sense idea of life-cycle of a product. This idea is well elaborated in system engineering, but one can detect its existence also outside engineering, for instance in archeology. The initial claim is that if we observe closely all events in which a given artefact participates, we can discover certain distinctive phases in its “life”.^6^ In engineering the standard ISO/IEC 15288 *Systems and software engineering – System life cycle processes* distinguishes six main phases or stages therein:

1. Concept – when the requirements for the artefact to be designed are defined and analysed;
2. Development – when the artefact is designed;
3. Production – when the artefact comes into existence;
4. Utilisation – when the artefact is used;
5. Support – when the artefact is serviced;
6. Disposal – when the artefact is retired, archived or disposed.

In archeology French prehistorians developed the so-called chaine operatoire concept for the purpose of lithic analysis, which concept seems to be deceptively similar, *ceteris paribus*, to the idea of ISO 15288 system life-cycle:

Technology is not typology. It takes into account the entire lithic material without preferentially isolating what we choose arbitrarily to call “tools”. It places each item in the sequence of technical actions beginning (after its conception and prior contemplation) with the raw material and ending with the abandonment, the “death” of the tool assemblage. Even when fragmented into thousands of microliths and “debris”, a lithic assemblage always forms a coherent whole bound together by a methodical scheme. (This is an English translation from (Bar-Yosef and Peer 2009) of one of the opening passages in (Tixier et al. 1980))

My notion of artefact is a simplification of the ISO/IEC 15288 construal. Instead of six stages I will consider only five with slightly different labels:

1. design (De)
2. production (Pr)
3. utilisation (Use)
4. service (Ser)
5. disposal (Di)

These five processes will be interpreted as attributes of frame representing artefacts. I will develop this idea in two stages: first introducing basic and then extended frames.

### Basic Frames

**Fig. 3:**
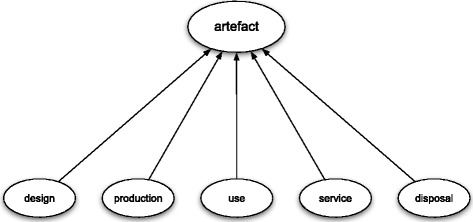
Main basic frame for artefacts

The main basic frame for an artefact consists of the five aforementioned attributes – see Fig. 3 – each of which has a binary set of values {0,1}.^7^ If an attribute has the value 1, this means that the history of an artefact comprises a process that instantiates this attribute. Accordingly, the value 0 represents a situation in which the artefact’s life does not comprise any such instances. I will call such frame the *main basic frame for artefacts* and its variants will be referred to as *basic variants*.

Strictly speaking, basic (and also extended) frames directly represent the *histories* of artefacts and artefacts are represented indirectly as object participating in those histories. Thus, strictly speaking, the five attributes of design, production, use, service, and disposal are attributes of the histories of artefacts and not of the artefacts themselves. The latter are characterised by the attribute of being designed, being produced, etc. Consequently, all my claims regarding frames representing artefacts are to be interpreted in this manner.

In principle any connected subgraph of the main basic frame may count as a variant of this frame, however, there are certain philosophical limitations on this combinatorial variety, i.e., not every subgraph of the main basic frame represents an artefact. However brushing aside, for a moment, these “excluded subgraphs”, one can say that an artefact is an entity that was either designed or produced or used or serviced (maintained) or disposed.

Let me now introduce a bit of notation to speak about main basic frames and their subgraphs. A frame subgraph will be represented as a sequence of acronyms of its attributes. The order of this sequence follows the order of attributes in the enumeration above because the basic frames do not carry any information about the temporal order of their attributes. This implies, among other things, than no sequence contains more than one occurrence of the same attribute abbreviation. Thus, for instance, DePrUse will represent a variant of the basic main frame, which represents those artefacts that are/were designed, produced, and used. Each such sequence is meant to provide a complete characteristics of a given artefact type, so DePrUse represents those artefacts that are/were not serviced or disposed. This implies that the categories I define in terms of frames are pairwise disjoint, e.g., there is no artefact that both DePrUse and DeUseSerDi represent.

Now consider the class of subgraphs of the main basic frame that contain the design attribute, but do not contain the production attribute. Consider for example the “would-be” frame DeUse. It is to represent those objects that were designed and used, but were not produced, i.e., which did not make it to existence. The problem with them is obvious: you cannot use an object unless it exists and the frame DeUse pretends to represent such objects. Consequently, the following subgraphs of the main basic frames are *not* considered here as its variants: DeUseSerDi, DeUseSer, DeUseDi, DeUse, DeSerDi, DeSer, and DeDi.

Among the remaining subgraphs there are three other disputable cases: SerDi, Ser, and Di. Consider the first case. It represents those objects that are somehow being serviced, i.e., maintained in a working condition, and disposed without being designed, produced or used. Since they were not produced, only the so-called natural objects like (some) stones and (some) trunks may fall under SerDi. Suppose that a certain stone is of this kind. If this stone were an artefact of the type UseSerDi, we could make up a story that someone found it, used it as an altar for several years, during these years he removed moss and lichen, and finally disposed it during a crisis of faith. But what kind of story can be told when we want to describe an object that was only serviced and then disposed? The main problem here is how to make sense of the operation of maintenance. If the object in question is neither designed nor produced nor used, how can we meaningfully claim that we maintain it? If it were designed and produced, we could claim that maintenance means restoring some of its designed and/or produced features. If it were only used, we could claim maintenance means restoring some of its properties that are important for the way we use it. Without either design or production or use one cannot explain and justify the claim that a certain process is a maintenance operation with respect to an instance of SerDi. A similar argument disqualifies Ser as a variant of the main basic frame.

The case of Di artefacts is different. Now the problem is that if Di were a frame for artefacts, then any object we intentionally destroy would become an artefact just because it was destroyed thereby. One could even argue that it becomes an artefact at the end of its disposal, i.e., it becomes an artefact when it ceases to exist. These consequences seem to overstrain our common-sense intuitions concerning artefacts or even objects in general.

The above considerations may raise a doubt whether De is a basic frame, i.e., whether it represents any artefact. One may say, for instance, that De concerns nothing else but a specimen or a blueprint for an artefact that will not make it to the existence. It is not thus a frame for an artefact because there is nor will be any. Even if it is conceded that this frame represents something, this something is not an artefact, so you cannot use it to make a cup of tea or to calculate the compound interest on your mortgage. Still I claim that De is a genuine basic frame. The reason for this claim has to do with Wartofsky’s classification of artefacts – see (Wartofsky 1979, p. 202–209). Due to the lack of space I refer the reader to a summary from (Susi 2006):

Primary artefacts are ones used directly in production, such as axes, needles, bowls, etc. Secondary artefacts are internal and external representations of primary artefacts, and they are created and used “in the preservation and transmission of the acquired skills or modes of action or praxis by which this production is carried out” (p. 202). As such, secondary artefacts are representations of these modes of action. Representing a mode of action also means that these artefacts are related to conventions, as in rules and norms. Tertiary artefacts are imaginary artefacts such as art or free play or game activity. Such artefacts have lost their original role of representation, since they have become “abstracted from their use in productive praxis” and from their “direct representational function”. (Susi 2006, p. 2211)

If we agree that Wartofsky’s “tertiary artefacts” are *bona fide* artefacts, then among all frames only the De may extend our notion of artefacts to include them. After all, most of the tertiary artefacts are designed in such a way not to make it to the existence. Similarly, among the “secondary artefacts” there are those that can be represented only by this type of frames. For instance, the category of such entities as plans (and also strategies, methods and the like) include both plans that were implemented and those that were never realised. It seems that the former do not differ, *qua* plans, from the latter. And again only De frame may represent the latter. Needless to say, artefacts represented by De are borderline cases of artefactuality, but the very idea of family resemblance is to include also such atypical instances.

As a result, we are left with 21 variants of the main basic frame. This frame and its variants constitute the “abridged version” of my family resemblance notion of artefact. Although this notion is extensionally capacious, including as many borderline cases of artefacts as possible, it does not include all of them. If you use a tiled stove to heat your apartment, then the ash and the soot it produces are not artefacts in the sense of this conception. On the other hand, when using this stove you also *intend* to obtain a large quantity of ash, then the ash produced thereby becomes an artefact. Generally speaking, the view elaborated in this paper implies that no object that is an unintended result (of a process of some kind) is an artefact.

### Extended Frames

Let me now expand the notion of basic frames by elaborating the specific structure and content of each attribute: De, Pr, Use, Ser, and Di. Since a proper characterisation of each of them would, in fact, require, a separate paper, my characterisation will be rather cursory.

Each attribute is construed here as (a representation of) a process (or a perdurant, if you like) (i) in which some agent or agents actively participate(s) as its actor(s) and (ii) whose beginning and end are characterised in terms of certain objects (i.e., endurants) and properties.^8^ The latter will be referred to as “input” and “output” objects and properties to a process in question.^9^

Besides the “usual” ontological difference between objects and their properties, I will employ an additional distinction. Namely, an object that is an input to a process is assumed to exist (at least) at the moment at which the process starts. Similarly, for output objects: an object that is an output to a process must exist (at least) at the moment at which the process ends. On the other hand, both input and output properties (to a process) carry no existential commitment. More precisely speaking, a property at an input (*resp*. output) to a process either exists at the beginning (end) of the process, i.e., is exemplified at that time by some object, or is an object of someone’s intention (at that time), i.e., is being desired or intended (by someone) to exist. For instance, when a customer specifies his or her requirements, I will represent these requirements as a set of intended, but unexemplified properties.^10^

**Fig. 4:**
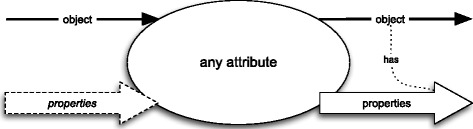
Attributes as blackboxes

Each attribute may be presented as a blackbox diagram – see Fig. 4. The block arrows represent sets of properties and “line-thin” arrows represent single objects. In order to distinguish “exemplified properties” from “intended” ones I will use the dashed block arrows (and the italic font) for the former and the solid block arrows for the latter. Each block arrow represents a non-empty set. If a set of properties, which is represented by a block arrow in a diagram, contains only those properties that are exemplified by an object, which is represented by a “line-thin” arrow, then the former and the latter are connected by a dotted line labelled “has”.

#### Design

**Fig. 5:**
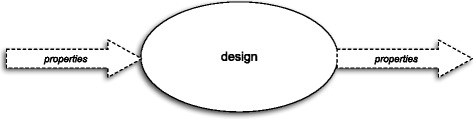
Design attribute as a blackbox

The design attribute represents a process that is characterised by two sets of properties: one at its input and one at its output, but has no “input” nor “output” object – see Fig. 5. The properties at the input are those features (i) that are for some reason desired or required (to be exemplified) and (ii) that are such that the respective artefact will assist its users to realise or exemplify those features. Thus, they constitute the goals or the rationale for the sake of which the respective artefact is going to be produced. In other words, they are those properties to which one will refer when asked “What is … for?”, where the dots stand for the artefact at stake. Obviously, such requirements can be entertained both by those agents who are directly involved in the artefact’s life (e.g., designers, producers, users, etc.) and by those who remain “external” to the whole process. Usually, the “input” properties are to be exemplified by objects different from the artefact itself. At the time of design phase they do not need to be exemplified by any real object, but they need to be desired (or intended) to be exemplified. On the other hand, the “output” properties to the process of design are those properties that are to be exemplified by the artefact itself. They are similar to the “input” properties with respect to their existence: at the end of the design phase they are not exemplified by anything, but they are desired (or intended) to be exemplified. In contradistinction to the “input” properties, the agent who intends them to be exemplified may be the designer of the artefacts, i.e., the agent who actively participate in the process of design.

**Fig. 6:**
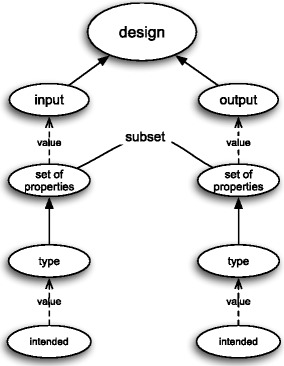
Frame for design attribute

Figure 6 depicts the design attribute as a frame in the sense of Barsalou. The information conveyed by this frame can be summarised as follows. The frame for the design attribute has two attributes: input and output. Each attribute may have a value that is a set of properties. Both sets of properties also have one attribute that specifies their type. And again in both cases this type characterises them as sets of intended properties. Needless to say, the same information is conveyed by Fig. 5, albeit in a less formal way.

#### Production

**Fig. 7:**
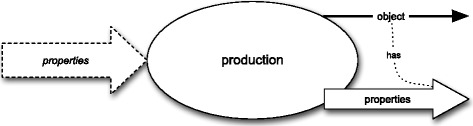
Production attribute as a blackbox

The production attribute represents a process characterised by two sets of properties: one at its input and one at its output, and by an “output” object, i.e., the artefact itself – see Fig. 7.

Any production process culminates in the creation of a new object. I assume that this event goes beyond a simple modification of existing objects and genuinely increases their number. The “input” properties to this process are those that the producer of the artefact, i.e., this agent who actively participates in production, intends to exemplify in the artefact. The output properties *correspond to* those “input” properties that are successfully exemplified at the end of this stage. Thus, for each property from the output set, which is actually exemplified by the artefact, there exists an “intended”, unexemplified property in the input set. Since I want to keep the ontological commitments of my conception minimal, I do not claim that the output properties *are* those “input” properties that are successfully exemplified. I do claim that there exists an injective mapping from the output set to the input set: for each “exemplified” property from the former set there exists exactly one property from the latter set that was intended to be exemplified. If you believe that there are unexemplified properties, which simply get exemplified at some stage in their existence, you could say that this mapping is the relation of identity. In any case it follows then a process is an act of production only if the producer managed to exemplify at its end at least property he or she intended to at its beginning.^11^

Finally, let me emphasise that a process of production need not be preceded by a design phase. When a production agent starts his or her activity without considering any goals or rationale for the artefact to be produced, but he or she simply intends that a certain set of properties should be exemplified and this intention is not a result of an earlier process of deliberation, then this situation is a case of a life-cycle that starts at the production phase. Obviously, it is possible that this production phase is the only phase in the life-cycle – this happens when the produced artefact is “abandoned”, i.e., when nobody will use, maintain, and dispose it.

#### Use

An act of use is characterised (i) by means of an object being selected as a tool or instrument and (ii) by means of its properties because of which it is selected as such. Obviously, the object is the artefact itself, which exemplifies the selected properties. I assume that the selection of the object and its properties happen at the beginning of each use phase.

There is a clear difference between design and production, on the one hand, and use, on the other. The difference at stake amounts to whether a phase may occur more than once in a life-time of one artefact. If you assume the principle to the effect that no entity can have two beginnings in existence (see the famous quote in (Locke 1689, II.27.1)), then this principle guarantees the uniqueness of the production attribute within a single frame for one artefact. The case of the design attribute is slightly different. In principle it may happen that this phase is composed of a number of separate activities that do not overlap either in space or time. However, since my artefact frames are not complex enough to account for such differences, e.g., since they do not represent time, I will coalesce all such separate events into one design process.

**Fig. 8:**
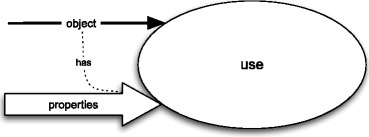
Use event as a blackbox

On the other hand, the life-cycle of an “average” artefact contains numerous uses thereof. Each such use may be different from the others not only because of its spatio-temporal location, but also because of the properties that each “use agent” selects. Since it is usually impossible to learn about all such cases of use for a single artefact, the use attribute is to represent all of them collectively. But then this difference of attributes has an important consequence with respect to the way in which we should represent frame attributes by means of input and output properties and objects. Namely, for the “unique” attributes, such as design and production, these representations are also unique, i.e., we can speak both about *the* “input” (*resp.* “output”) properties of a design attribute and about *the* “input” (*resp.* “output”) properties of a frame to which this attribute belongs. On the other hand, since the use attribute of a frame represents various instances of use, it makes little sense to speak about the “input” properties of the use attribute. Therefore, although each case of use can be depicted as in Fig. 8, the attribute of use in a frame for an artefact will be represented without any inputs.

Similar remarks can be made about the two subsequent attributes: Ser and Di. The former represents all cases of service activity that the artefact undergoes during its lifetime. The latter represents an event of its destruction, which is unique once you accept the counterpart of Locke’s principle: no entity can have two ends in existence. Incidentally, let me note that I do not exclude the possibility that the object has been destroyed during its use.

It is quite straightforward that use events are different from production phases in that when a use event for an artefact *x* commences, *x* exists, while when a production phase (for *x*) is being initiated, *x* does not exist yet. Thus, the difference between, say, an artefact represented by the frame Use and an artefact of the frame PrUse is that the former (e.g., a stone) comes to existence without human intervention, but happens to be used, and the latter (e.g., a sculpture that is created “on the fly”) is a result of some intentional production process, which result at some later stage of its life time is also used. So, when you wrap a piece of carp fish in a banana leaf, this leaf is an instance of Use, but when you build a food container out of banana leaves and then use it as a kitchen utensil, then this container is not an instance of Use but of PrUse.

#### Service

**Fig. 9:**
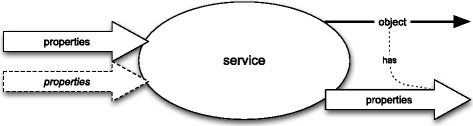
Service event as a blackbox

An instance of service activity is a process that can be characterised by two sets of properties and the artefact as inputs – see Fig. 9. One set contains the properties actually had by the artefact. The other set contains those properties that the agent wants to restore in the artefact. It is the latter set that was the reason for my downgrading of certain subgraphs of the main basic frame. Now I can say that the input set of “unexemplified properties” for a service event is either

1. a subset of the De output set of properties;
2. a pseudo-subset of the Pr output set of properties;
3. a pseudo-subset of the set of properties that were selected by a user of this artefact at the beginning of one of its earlier events of use.

These three cases represent three different possibilities of the goals that the service agent may wish to achieve. In case 1 the service process aims at recovering those properties that were envisaged at the design phase of the artefact in question. The case 2 covers the situation where the focus is on those features that actually came out of the production phase. Finally, the service may also be aimed at re-establishing those properties that were selected by a user of this artefact even when they do not coincide either with the “designed” or “produced” features. The output set of properties in a service event is a pseudo-subset of its input set of “unexemplified” properties.

#### Disposal

**Fig. 10:**
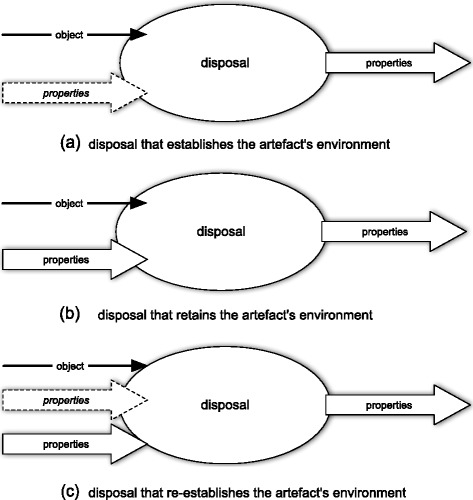
Three types of disposal attribute

The disposal attribute represents a process that culminates in the destruction of the artefact. In contradistinction to other processes, in particular in contradistinction to the process of use, the agent who is responsible for this activity (“disposal agent”) considers both the artefact itself and a set of properties to be exemplified by objects *other* than the artefact. The disposal agent either attempts to exemplify them (Fig. 10a) or attempts to retain those already exemplified (Fig. 10b) or both (Fig. 10c). Usually, the properties in question are to be exemplified by the artefact’s environment, i.e., by a complex system of interrelated entities that includes the artefact. Which properties of this environment are considered by the disposal agent depends on particular circumstances, but in general they depend on the socio-technical context in which the artefact’s life-cycle is involved, in particular on the current legal system in which the artefact’s designers, producers, users, etc., are involved.^12^

Loosely speaking, an act of disposal of an artefact is a process of its destruction that takes place in the artefact’s environment. Each process of this kind does not amount just to the artefact’s ceasing to exist. An act of disposal is an intentional activity of certain agent who intends to achieve two objectives:

1. that the artefact ceases to exist and
2. that some other objects in its environment exemplify certain properties.

The first type of disposal attribute (Fig. 10a) covers those situations in which the disposal agent attempts to create a new environment after the artefact ceases to exists. Obviously, the output set of properties (of this attribute) would then be a pseudo-subset of the set of input properties. The second type of disposal attribute (Fig. 10b) covers those situations in which the disposal agent attempts to retain some aspects in the artefact’s environment. Now, the output set of properties would be a subset of the set of input properties. The third type, probably the most common, combines the previous cases.

The 21 variants of the main basic frame can be defined for extended frames in an obvious way. On top of this extension one can further distinguish:

1. for each frame that contains the Di attribute, its three variants that correspond to the three cases of the disposal activity depicted in Fig. 10;
2. for each frame that contains both De and Pr, its two variants that correspond to two possibilities:
  - the input set of properties from Pr is a subset of the output set of properties from De;
  - the input set of properties from Pr is *not* a subset of the output set of properties from De;
3. for each frame that contains both De and Di, its two variants that correspond to two possibilities:
  - the input set of properties from Di is either a subset (the subcase for situation depicted in Fig. 10a) or a pseudo-subset (the subcase for Fig. 10b) of the input set of properties from De;
  - the input set of properties from Di is neither a subset nor a pseudo-subset of the input set of properties from De.

The case 2a represents the “standard” situation in which the properties that were designed at an earlier stage in the life-cycle of an artefact guide its production process. On the other hand, the case 3a corresponds to the situation where the initial design requirements envisage not only the rationale for the artefact at stake but also the details of its disposal.

The relations of being a subset and of being a pseudo-subset are considered here as the structural invariants of the extended frames. Moreover, those that are mentioned in the above enumeration are considered facultative, i.e., they may not occur in some extended frames.

**Fig. 11:**
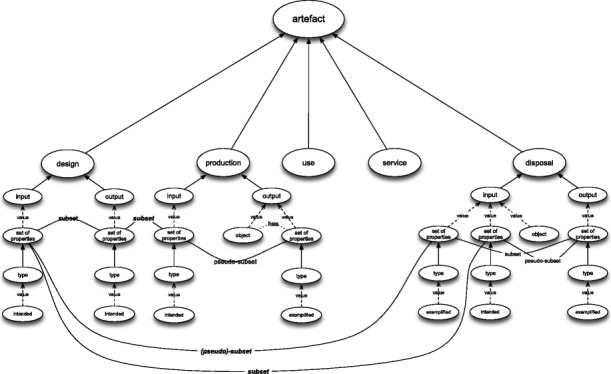
The main extended frame for artefacts

In sum there are 81 extended frames for artefacts with the main extended frame depicted in Fig. 11. Taken together with the above explanations they constitute the full version of the family resemblance notion of artefact.

## Multi-faceted Categorisation of Artefacts

Does the above conception shed any new light on the problem of artefact categorisation?

First, note that it provides a top-level categorisation of artefacts since each frame may be interpreted as a top-level category of artefacts. Thus, for instance, DePr will collect all artefacts that are designed and produced, but were never used nor serviced nor disposed, and UseDi will accumulate all natural entities, i.e., objects that were not produced, that happen to be used and then disposed. Any categorisation at this level of generality is of little use unless it is employed to classify entities of similar generality, e.g., the concepts of artefacts themselves. Table 1 shows how basic frames may be used for this purpose. Namely, I compare there the extensions of the concepts as defined in philosophy (Baker 2007, p. 52–53), (Dipert 1993, p. 23–33), (Hilpinen 1993), (Houkes and Vermaas 2010, p. 158–160), Artificial Intelligence (Borgo and Vieu 2009), and in engineering design (the definition developed in the Department of Knowledge Systems of Osaka University by R. Mizoguchi and his associates), to the extensions of my 21 basic frames. The reader can find a more detailed description of the six aforementioned conceptions in Appendix A below.

**Table 1:**
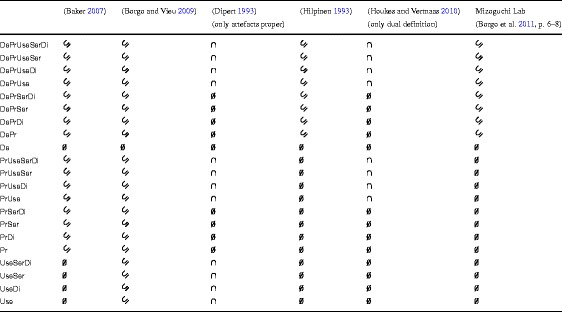
Family resemblance across concepts of artefacts

The results of comparison are rendered by means of three symbols:

- $\subseteq $ – when the extension of a basic frame is a subset of the extension of a respective concept;
- $\cap $ – when the extension of a basic frame properly overlaps the extension of a respective concept;
- $\emptyset $ – when the extension of a basic frame does not overlap the extension of a respective concept.

My comparison is based on a number of assumptions concerning the interpretation of the definitions in question. In particular, I assume

1. the conditions listed in (Baker 2007, p. 52–53) imply that the history of an artefact always involves an event of creation of a new object, i.e., the artefact itself. This event may be preceded by an earlier design activity and may be followed by subsequent events of use, service, and disposal;
2. that each event that is represented by De, Pr, or Use is an event of intentional selection (IntentionalSel) in the sense of (Borgo and Vieu 2009);
3. that the notion of modification used in (Dipert 1993) subsumes my Pr attribute and that not every artefact that falls under one of my basic frames exhibits “modified properties [that] were intended by the agent to be recognized by an agent at a later time as having been intentionally altered for that, or some other, use” (Dipert 1993, p. 29–30);
4. that (Hilpinen 1993) makes a distinction between an event of selection of desirable properties (my De attribute) and an event of implementation of those properties (my Pr attribute) and that the history of an artefact requires both;
5. that the notion of design employed in (Borgo 2011, p. 6–8) by R. Mizoguchi and Y. Kitamura corresponds to my De frame and that their notion of production corresponds to my Pr frame;
6. that the so-called “dual definition” from (Houkes and Vermaas 2010, p. 158–160) implies that a fully-fledged artefact both undergoes a proper production process and is involved in one or more use events and that there are such use events that do not fall under their notion of use plan.

If one were to translate those definitions into the current framework, then one would get the following descriptions:

1. Baker (2007) selects the production phase as the essential aspect of artefacts;
2. Borgo and Vieu (2009) select any phase without differentiating between them – with one exception: De frame does not represent artefacts in their sense
3. Dipert (1993) selects use as the essential aspect;
4. Hilpinen (1993) selects the phases of design and production;
5. R. Mizoguchi and his associates also select design and production;
6. Houkes and Vermaas (2010) select production and use.

Needless to say, these “translations” do not pick up the most salient features of the conceptions in question as they oversimplify them significantly. If you compare them to the originals (or even to the abridged descriptions thereof in the appendix), you will clearly see the difference in perspective. It is this difference that makes most of the distinctions that are available in the life-cycle approach superfluous in the context of those conceptions. For instance, although UseSerDi, UseSer, UseDi, and Use are four different frames that represent four (extensionally disjoint) categories of artefacts, for (Dipert 1993) all of them have the same status. On the other hand, the distinction between UseSerDi and PrSerDicould make sense for him.

Secondly, the family resemblance notion provides a new set of terms for the debate about artefact categorisation. The current state of this debate is dominated by the “categorisation-by-function” camp and its opponents. According to the former camp whether a certain artefact belongs to a given category of artefacts depends solely on its functions (e.g., (Baker 2007)). The dissenters to this view, while acknowledging a role of functions in artefact categorisation, point out to other aspects, like the intentions of the artefact’s designer (Vaesen and van Amerongen 2008) or a use plan for this artefact that was envisaged during its design – see (Houkes and Vermaas 2010).

The perspective I outlined above substitutes most of these concepts with a batch of new ones. Namely, that whether a certain artefact belongs to a given category may depend on:

1. its properties at the design phase:
  - those that come out of this phase as its intended properties;
2. its properties at the production phase:
  - those that are planned to be exemplified
  - those that are actually exemplified;
3. its properties at one of its use phases:
  - those that a user of the artefact considers at this phase while he or she selects it as a tool;
4. its properties at one of its service phases:
  - those that are planned to be restored (re-exemplified) at this phase
  - those that are actually restored after this phase is finished.

In principle artefact categorisation may be extrinsic to the artefact itself and may depend on the properties of other objects. Then the above list can be extended with

5. those properties that constitute the rationale for the creation of the artefact at the beginning of the design phase (the “input” properties of the design attribute);
6. those properties that the disposal agent intends either to establish or to maintain in the artefact’s environment at the beginning of the disposal phase;
7. those properties that are actually established or maintained in the artefact’s environment at its disposal.

Under certain conditions, which I mentioned above, this variety of categorisation facets may be slightly simplified by:

- 2a being reduced to 1a
- 4a being reduced to 2a or to 3a
- 6 being reduced to 5

Then the aspects of artefact categorisation can be arranged in a binary taxonomy:

1. intended properties
  - “output” properties of De
  - “input” properties of De
2. exemplified properties
  - “output” properties if Pr
  - properties considered by a user (of an artefact) at a use phase
  - properties actually restored after a service phase is finished
  - “output” properties of Di.

The reader should bear in mind that some properties from 1a and all properties from 2d are extrinsic to the artefact, i.e., they are exemplified not by the artefact but by some other objects (from the artefact’s environment).

The above conception introduces a new perspective on the problem of artefact categorisation. Instead of analysing the ontological status of artefacts as if they did not evolve in time or instead of focusing at one, allegedly significant, phase in their history, I recommend tracing the changes of this status throughout their life-cycles. This perspective brings forward two groups of properties that were neglected in the debate on artefact categorisation. The first group involves those properties that are either intended to be restored or are actually restored in an event of artefact maintenance. The other type concerns those properties that are either restored or established during the disposal of the artefact. So instead of classifying artefacts with respect to their designed properties one can classify them with respect to those properties that are important from the point of view of maintenance or disposal. At the same time the perspective undermines the significance of the functions vs non-functions distinction in this debate. Although the most appropriate “place” for artefactual functions appears to be at the output of the design phase, one can find examples of artefacts whose functions come into existence at the input of the production phase or at the input of a use or service event. The former cases concern those situations in which the production specification diverges from the design specification, e.g., when a manufacturing agent decides that it is not feasible to implement one of the designed properties or that it is feasible and desirable to implement a property that was not envisaged in the design. On the other hand, when you use a certain artefact to achieve a goal that is not among its goals defined in the design or production phase, you are involved in a situation of the latter kind. So when you use a hammer as a paperweight, a new function of this hammer comes into existence.

## Discussion

My account of artefacts represents them by means of a family resemblance concept. Even if some details do not correspond to how we actually conceptualise and categorise them, the psychological research on concepts and categorisation makes it improbable that the “real” concept of artefacts may be captured by means of a unique set of necessary and sufficient conditions. The classical approach to concepts seems to be closed for those who intend to engage rather in descriptive than in prescriptive philosophy. The device of family resemblance interpreted as a frame in the sense of L. Barsalou brings my proposal closer to the actual activity of artefact categorisation.

On the other hand, the above considerations are meant to provide only a *regulatory* definition of artefacts, which:

[…] while modifying the original vague meaning of a word, sharply delineates its extension, but takes into consideration the original vague boundary of its extension, as suggested by the original meaning. (Ajdukiewicz 1974, p. 75)

My aim was thus not to represent our concept of artefacts in all detail. First, the linguistic variety of the term “artefact” and its derivatives^13^ and the differences between philosophical theories of artefacts cast a doubt whether there is a single concept that covers the whole domain of artefacts. Even if it turns out that there is, then it seems that the concept in question, as opposed to the so-called basic categories, is too abstract and too vague to be stable both across different subjects who use it and different times when it is used.

Being located between the two extremes of the descriptive and the prescriptive analysis my conception is to provide a new methodological perspective on the domain of artefacts with respect to the problem of adequate characterisation of its members. The novelty in question concerns the fact that instead of propounding a single, crisp notion of artefact, I provide a family of such notions, which can be applicable in various conceptual circumstances. In particular my conception is capable of accommodating different epistemological interests of those who aim to categorise artefacts. As the research surveyed in (Malt and Sloman 2007) attests, an event of artefact categorisation usually depends on the cognitive task to which it is assigned. This dependence involves, among other things, an appropriate selection of those properties that are relevant for this task and which are thus the basis for this categorisation event. My conception attempts to delineate the range of possible selections and to classify them. Moreover, it propounds two “new” types of properties suitable for this purpose. In particular, the “disposal” properties seem to be a realistic alternative to the traditional bases of artefact categorisation.

Nevertheless, experimental validation of the above model would be both a stimulating and instructive challenge if it did not exceed the research competencies of the author of this epistemic artefact.

## Appendices A: Six Conceptions of Artefacts

### A.1 Lynn R. Baker’s Constitution View on Artefacts

The conception of artefacts formulated and defended by L. Baker in (Baker 2007, p. 52–53) can be summarised by the following four conditions for x’s being an artefact: (A1) x has one or more makers, producers, or authors. Designers and executors of design […] are authors. (A2) x’s primary kind (its essence, its proper function) is determined in part by the intentions of its authors. (A3) x’s existence depends on the intentions of its authors and the execution of those intentions. (A4) x is constituted by an aggregate that the authors have arranged or selected to serve the proper function entailed by the artefact’s primary kind. So according to this conception an artefact is an object constituted by other objects which were selected and/or arranged to serve the proper function which is established by the designers or those who embody/realise the artefact’s design. Note that the actual kind of an artefact is determined solely by its proper function: What proper function an artefact has determines what the artefact most fundamentally is – a boat, a jackhammer, a microscope, and so on. And what proper function an artefact has is determined by the intentions of its designers and/or producers. (Baker 2007, p. 52)

### A.2 Artefacts in Formal Ontology—the Case of (Borgo and Vieu 2009)

S. Borgo and L. Vieu express their conception of artefacts within the context of formal ontology, i.e., they express their views in the language of formal logic by means of axioms and definitions, so this conception is a formal theory in the sense of logic. To make the matters worse, this theory employs the terminology of another formal theory, by the name of dolce, which is a foundational applied ontology build for the purposes of knowledge representation in Artificial Intelligence. dolce describes its categories, including those used in (Borgo and Vieu 2009), by means of several dozens of axioms and definitions, so the adequate characterisation of this conception is not possible here. However, if one brushes aside the nitty-gritty details of its formalism and focuses on its philosophical insights, one may come up with the following summary.

First, Borgo and Vieu establish the ontological category of artefacts as that of physical endurants, i.e., each artefact is an entity that endures in time and is located at some region in space. As a result, although there might exist artefactual properties and processes, they will be not classified as artefacts.

Secondly, artefacts are those physical endurants that were created by some agent who is capable to entertain such intentional attitudes as beliefs, desires, and intentions. The notion of creation is rather idiosyncratic here because it is constrained to the intention to create an entity with a certain desired property, so it does not require any action of physical modification. I create an artefact when I select a certain physical object and attribute certain capacities thereto. Although the existence of the physical object I selected precedes my act of selection, the actual capacities of this object may be divergent from the capacities I attributed to it. For instance, when I simply select a certain pebble and ascribe to it the capacity to produce electrical energy, by means of those two mental actions I created a new artefact, say, a dynamo. Note that the artefact is not the selected object, but the entity that latter constitutes, e.g., the dynamo, which is the artefact, is constituted by the pebble, which is the object I selected.

### A.3 R. Dipert’s Grades of Artefactuality

Instead of providing us with one definition of artefacts R. Dipert propounds four interrelated notions: that of tools, that of instruments, that of artefacts proper, and the notion of artefactual side effects. The rationale for this kind of approach can be summarised as follows: This is an attempt to classify, or form grades of kinds of, near-artifacts, in the hope of gradually highlighting the salient features of artifacts themselves. These near-artifacts consists of what I call “instruments”, “tools”, and an interesting residue of objects that are in some sense artificial but are not tools (and typically are not instruments either), such as waste products from intentional activity. (Dipert 1995, p. 121) It seems that the minimal grade of artefacthood is exhibited by artefactual side effects of our intentional activity, in particular when we are not aware of their existence or even are not able to know them – see (Dipert 1993, p. 33–37). A more “artefactual” category is constituted by tools. A tool is an entity such some agent used it to achieve a certain goal. An instrument is a tool that was intentionally modified so that its user may achieve this goal.^14^ Finally, an artefact is a tool such that besides its modified properties it exhibits a number of other properties, which Dipert labels as “communicating properties”, due to which a user of this tool is able to recognise that this tool was intentionally modified to assists him or her to achieve a certain goal. (Dipert 1993, p. 23–33 )^15^

### A.4 R. Hilpinen on Artefacts and Their Authors

Hilpinen develops his account of artefacts in parallel with his conception of authorship. An artefact is construed as an entity that bears the relation of authorship to some agent, i.e., to its author. This simple statement, which is labelled in (Hilpinen 1993) as axiom A1, is elaborated by him by means of a number of further axioms that specify the most relevant aspects of this relation. The phrase “the object’s specification of production” refers to the intentional content that guides the agent’s production of the object (Hilpinen 1993, p. 158).

A2 If an object is authored by an agent, then there exists a type (sort) such that the type is included in the object’s specification of production and the existence of the object depends of the agent’s intention to produce an object of this type.
A3 If an object is authored by an agent, then there exist certain properties such that the object exhibits them and this fact depends on the object’s specification of production.
A4 If an object is authored by an agent, then there exists a type (sort) such that the type is included in the object’s specification of production and the object instantiates this type.
A5 If an object is authored by an agent, then there exist certain properties such that if the object’s specification of production had included them, the the object would have exhibit them.
A6 If an object is authored by an agent, then there exists a type (sort) such that the type is included in the object’s specification of production and the agent accepts the object as instantiating this type.
A6 If an object is authored by an agent, then the agent accepts the object as a satisfactory realisation of the object’s specification of production.^16^

### A.5 Man-made Objects in Action

The account of artefacts developed in (Houkes and Vermaas 2010) should be interpreted against the background of their ICE theory of artefactual functions or even more broadly against the context of the use plan framework to represent artefacts and their functions. However, the lack of space allows me to spell out only the final result, i.e., the definition of artefacts, which is dubbed in (Houkes and Vermaas 2010, p. 158–160) as “dual definition of artefacts”: An object *x* is an artefact *a* of type *t* if and only if: (1) *x* has been intentionally produced by an agent *m*; and (2) *x* is manipulated in the course of executing a specific use plan *p*, which is designed, communicated and evaluated in accordance with the use plan analysis and designing. (Houkes and Vermaas 2010, p. 158) As the reader will probably anticipate, this account combines two other definitions. The first, which has become condition 1, represents artefacts as those objects that were produced in the course of an intentional activity of some agent. The second, which corresponds to condition 2, describes artefacts as objects that are used in the course of a different kind of intentional activity. Combined together the two definitions are claimed to be sanitized from some unwelcome consequences that each of them implies separately.

### A.6 An Engineering View on Artefacts

The view in question is claimed in (Borgo 2011, p. 6–8) to represent the category of engineering artefacts. It was developed by a group of researchers affiliated at the Department of Knowledge Systems of Osaka University for the purposes of knowledge representation in engineering design: A technical artifact a is a physical object created by an intentionally performed production process. The process is intentionally performed by one or more agents with the goal of producing the object a which is expected to realize intended behavior in some given generic technical situation. Although the intentionally performed production process aims at creating an object that will perform the intended behaviour, the object created by this process is an artefact even if it is not capable of performing this behaviour, i.e., a production process may turn out to be unsuccessful.

It is assumed here that an intentionally performed production process is composed of two phases: (i) design and (ii) manufacturing. The role of the former is to define a formal specification for the latter. The production process is usually followed by a use phase.

## Footnotes

1 M. Pelczar labels in (Pelczar 2000) this interpretation as standard.

2 The idea of family resemblance was also tested for applicability in Artificial Intelligence. (Carrara et al. 2011) attempt to show why the notion of engineering function may be represented as a family resemblance notion and what consequences this claim may bring for the formalisation strategies in applied ontology.

3 Barsalou represents an attribute of a concept by means of a single line that is labelled “aspect”.

4 As a matter of fact, Barsalou speaks about constraints in general and draws two orthogonal partitions thereof: (i) attribute constraints vs value constraints (ii) contextual constraints vs optimisations. Now, from the extensional point of view the notion of attribute constraint is subsumed by the notion of structural invariant since both the latter and the former express certain relationships between frames’ attributes even though their extra-logical properties are different, e.g.: “rather than being normative [as structural invariants are], constraints produce systematic variability in attribute values.” (Barsalou 1992, p. 37). Therefore, I mention only *value* constraints as a frame component.

5 Barsalou provides us with the example of the parthood relation, which is a structural invariant in great deal of frames. Following (Winston et al. 1987) he claims that parthood can be represented by a frame with four attributes: functionality, separability, homeomeronymy, and spatio-temporal extent.

6 The term “the life (of an object)” is to denote the mereological sum of all processes and events in which this object participates, i.e., its history.

7 Since each attribute has “the same” set of values, I do not include them in this figure for the sake of readability. For the same reason I drop the “aspect” label I used before.

8 Although I acknowledge the possibility that numerous agents may participate in such process (either on a cooperative or competitive basis), for the sake of simplicity I will use the singular form to refer to them and their activities.

9 I believe that the adequate ontological representation of artefacts requires the notion of state of affairs (see Garbacz 2009), but in this paper I follow the prevailing tradition that describes them in terms of properties or qualities – see (Baker 2007; Dipert 1995; Hilpinen 1993), among others.

10 I use the verb “exemplify” to denote the relation of exemplification as defined in (Lowe 2006, ch. 2).

11 If one property is intended to be exemplified and the other is “the same” property but exemplified, I will say that they are *equivalent*. If two sets *X* and *Y* of properties are such that for each property in *X*, there exists an equivalent property in *Y*, I will say that *X* is a *pseudo-subset of * the *Y*.

12 For example, the Directive 2000/53/EC of the European Parliament and of the Council of 18 September 2000 on end-of life vehicles specifies the requirements on the sites for storage and treatment of end-of-life vehicles and the sequence of treatment operations. These requirements are to guarantee that such end-of-life vehicle fluids as fuel, motor oil, gearbox oil, transmission oil, hydraulic oil, cooling liquids, antifreeze, brake fluids, battery acids, air-conditioning system fluids are separated, collected, and secured in impermeable containers so that the level of contamination of the soil, water, and atmosphere in the vicinity of these sites is kept at a minimum level. In this case the environment for a car to be scrapped is constituted by the storage and treatment site and the vicinity of this site.

13 The reader may find ample textual evidence thereof in (Parker 2010).
